# Social media and eating behavior in later life: psychological pathways, evidence gaps, and an age-inclusive research agenda

**DOI:** 10.3389/fpsyg.2026.1966068

**Published:** 2026-10-12

**Authors:** Dahai Hu, Weichuan Luo, Xueqi Wu

**Affiliations:** 1 School of Literature and Journalism, Xihua University, Chengdu, China; 2 Taylor’s Culinary Institute, Faculty of Social Sciences and Leisure Management, Taylor’s University, Subang Jaya, Malaysia

**Keywords:** aging, digital commensality, eating behavior, nutrition literacy, older adults, social media

## Abstract

Population aging is unfolding within a food environment increasingly organized through social-media platforms. This Mini Review develops a psychological framework linking social-media content and engagement to eating behavior in later life. A focused narrative search and targeted supplementary identification informed the selection of 26 synthesis sources, organized by population, exposure, and outcome into six evidence classes. Four pathways structure the synthesis: social connection and digital commensality; food information and nutrition literacy; social comparison, affect, and dietary self-regulation; and commercial cues and algorithmic curation. Socioemotional selectivity and information-processing theory anchor the relational and appraisal mechanisms linking digital encounters with meals. The evidence includes one direct older-user study with a dietary outcome, alongside eating-context studies, content audits, adjacent digital-health research, and broader-age bridging evidence. Later-life conditions shape exposure, processing, and response across the framework. Nutritional priorities include maintaining appetite, adequate intake, and function, alongside disease-specific dietary management. We identify research priorities that combine qualitative accounts of active content use with exposure measurement, longitudinal and experimental designs, and inclusive recruitment. The framework specifies testable relationships between digital experiences and eating outcomes while identifying the populations and conditions in which those relationships warrant examination.

## 1 Introduction

Population aging and the platformization of everyday life are converging transformations. By 2030, one in six people worldwide will be aged 60 years or older, and this population is projected to reach 2.1 billion by 2050 (World Health Organization., 2025). At the same time, food information, recipes, health claims, interpersonal exchanges, advertising, and support programs increasingly circulate through social-media feeds. These environments organize attention, social comparison, emotional responses, perceived norms, and opportunities to eat with or for others. They therefore constitute a consequential psychological context for eating behavior.

Eating in later life is embedded in a distinctive psychosocial ecology. A systematic review identified health beliefs, food-related control, mood, enjoyment, eating arrangements, social facilitation, and support as recurrent influences on older adults’ eating (Walker-Clarke et al., 2022). Ecological evidence likewise indicates that older-adult eating reflects interacting individual, interpersonal, community, and societal conditions (Montez De Sousa et al., 2022). Frailty, multimorbidity, appetite change, bereavement, retirement, mobility limitations, and living alone may alter dietary needs and the meaning of meals. Social networks are relevant to food choice and diet quality among frail older adults (Kim, 2016), while eating alone has been associated with depression, lower quality of life, and lower dietary diversity (Kimura et al., 2012). Social-media influences may therefore be consequential without being uniformly harmful or beneficial.

Older adults also engage with digital platforms under unequal conditions. Their social-media use is often motivated by maintaining family and friendship ties, whereas privacy concerns, perceived irrelevance, and usability problems can discourage participation (Newman et al., 2021). A food video shared by a family member, a livestream watched alone during dinner, an algorithmically recommended supplement claim, and a peer-support group for diabetes may all count as social-media exposure, but they involve different psychological processes. Treating them as equivalent minutes of “screen time” obscures whether a platform supplies companionship, credible knowledge, affective pressure, or commercial persuasion.

Research on digital food environments connects food content, online comparison, body image, advertising, and eating outcomes (Granheim et al., 2022; Wu et al., 2024). Applying these findings to later life requires attention to changing social goals, nutritional priorities, and opportunities for digital participation. Gerontological technology research offers complementary evidence on access, loneliness, cognition, and wellbeing (Newman et al., 2021). Integrating these traditions creates a psychological account of how digital food environments intersect with the circumstances of later-life eating.

This Mini Review asks how social-media exposure and engagement relate to eating, which later-life conditions shape these relationships, and which mechanisms offer priorities for empirical testing. We synthesize four pathways: social connection and digital commensality; food information and nutrition literacy; social comparison, affect, and self-regulation; and commercial cues and algorithmic curation. Our central argument is that age-conditioned configurations of content, engagement, and context provide a more informative analytic target than screen time alone. The framework connects these configurations to food choice, meal regularity, dietary variety, appetite, adequate intake, and dietary self-management.

## 2 Evidence identification and scope

We conducted a focused narrative search of Web of Science Core Collection and Scopus on 12 August 2026. Database discovery combined aging terms (e.g., older adults, elderly, later life), social-media and platform terms (e.g., Facebook, YouTube, WeChat), and eating terms (e.g., food choice, diet, nutrition, meals, appetite). Database records were restricted to English-language articles and reviews published in 2004–2026. Supplementary Material 1 provides the retained queries and search records. Targeted supplementary identification during revision added qualitative and theoretical sources and geriatric nutrition guidance; classic theory was eligible irrespective of the database date window.

Sources were selected purposively for their relevance to the four pathways and later-life eating, prioritizing studies of older users, later-life eating contexts, and nutrition content directed at or relevant to older audiences. The database counts in Supplementary Figure 1 document discovery and metadata processing, while the 26-source set describes the narrative synthesis. Broader-age studies and reviews were selected for their contribution to the four pathways or age-related evidence gaps. Social-media use solely for recruitment or data collection did not establish exposure evidence. Social media denotes networked platforms for creating, sharing, or exchanging socially circulated content; recommendation systems are a variable platform feature. Multicomponent digital-health evaluations were classified as adjacent when the contribution of their social-media exposure to eating outcomes was not separately evaluated.

Later life is interpreted primarily as age 60 years and older. Studies beginning at age 50 were retained as mid-to-later-adulthood evidence, with their actual age distributions reported in Supplementary Table 1. This distinction preserves evidence on transitions into later life while locating findings within the populations studied.

Each synthesis source was assigned one primary evidence role: D, studies evaluating a defined social-media exposure in relation to an eating behavior among older users; C, later-life eating context without social media; T, older-targeted or later-life-relevant content audits; A, adjacent media, digital-health technologies, or psychological and nutritional-status outcomes in mid-to-later adulthood; M, broader-age mechanism or contextual evidence; or G, youth-focused evidence used to identify an age gap. D outcomes include dietary choice, intake, variety, regularity, and dietary self-management. Body dissatisfaction, frailty, or nutritional-status scores alone were classified as A.

The synthesis contains 26 sources: D = 1, C = 5, T = 2, A = 10, M = 7, and G = 1. Five background, theory, guidance, and research-design references are identified separately in Supplementary Table 1 and excluded from these counts. These counts describe cited sources, including primary studies and reviews; Table 1 reports the sources primarily discussed in each pathway section, and Supplementary Table 1 records design, population, exposure, outcome, and source-level classification.

**TABLE 1:** Evidence supporting the four pathways and priorities for age-inclusive research.

Pathway and proximal process	Evidence in the pathway section	Key inference to test	Discriminating comparison
Social connection Social presence, relationship continuity, reciprocity	7 sources: C = 2, A = 3, M = 2; D = 0. Meal-context studies, digital-health evaluations, and commensality reviews.	Whether relational continuity and reciprocity connect digital contact with eating.	Compare synchronous meals, asynchronous exchanges, and passive viewing; measure meal regularity and dietary variety alongside social experience.
Food information Credibility appraisal, comprehension, applied literacy	7 sources: D = 1, T = 2, A = 2, M = 2. Direct dietary self-management evidence, content audits, literacy reviews, and broader-age mechanism and contextual evidence.	How source cues, presentation demands, and active appraisal relate to food decisions.	Vary source disclosure and cognitive load; assess credibility, comprehension, and choice separately, alongside health complexity and digital literacy.
Comparison and self-regulation Appraisal, affect, self-efficacy	5 sources: A = 3, M = 2; D = 0. Mixed-media, psychological, and nutritional-status studies; broader-age reviews.	Whether comparison-related changes in affect and self-efficacy precede changes in eating.	Use repeated measures of comparison goals, affect, appetite, intake adequacy, and dietary regulation; test reverse temporal ordering.
Commercial cues Familiarity, norms, commercial credibility	2 sources: M = 1, G = 1; D = 0. Digital food-environment and youth commercial-exposure reviews.	Whether repeated commercial exposure influences appraisal and subsequent food decisions in later life.	Combine platform-specific feed audits with experiments on repetition and disclosure; separate credibility, purchase intention, purchasing, and eating.

Counts assign each source to its primary pathway section: 21 sources are shown here and five further sources support the introduction and cross-cutting conditions. Secondary conceptual relevance to another pathway does not add to these counts. Sources are not independent samples. D, direct older-user exposure and eating behavior; C, eating context; T, content audit; A, adjacent evidence; M, broader-age mechanism or context bridge; G, age-gap comparator. Operational definitions and all source assignments appear in Supplementary Table 1. Classes identify evidence roles, not study quality.

The 26 sources comprise 15 primary investigations, 10 systematic or scoping reviews, and one conceptual review. Classification records a source’s role in the synthesis, while study design and the measures used determine the inference attached to each finding.

## 3 Four pathways from social media to later-life eating

The framework connects social-media exposure and engagement to eating through four partially overlapping psychological pathways (Figure 1). Their proximal processes comprise social presence and reciprocity; credibility appraisal and comprehension; comparison-related affect and self-efficacy; and familiarity, norms, and commercial credibility. Later-life conditions shape exposure, processing, and response throughout. Pathways can operate concurrently or in sequence: advice shared within a valued relationship engages social connection and information appraisal, while a sponsored food demonstration combines credibility cues and commercial persuasion. Age-conditioning refers to variation in pathway salience, direction, and accessibility across later-life circumstances. Table 1 links each pathway to its evidence base and a discriminating research priority.

**FIGURE 1:**
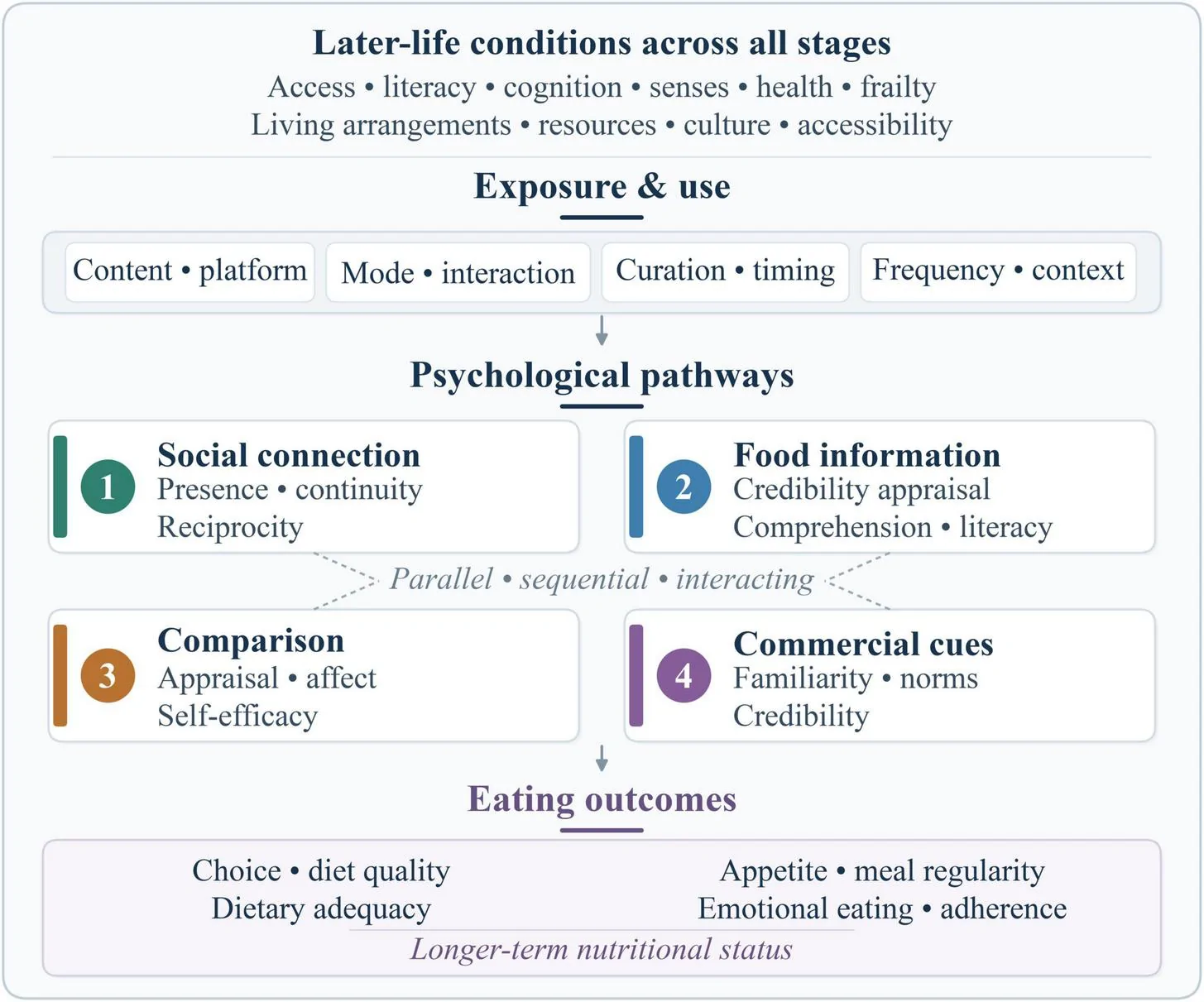
Age-conditioned pathways linking social media to eating behavior in later life.

The same encounter can also produce competing responses. A trusted peer’s restrictive-diet advice might strengthen social connection while conflicting with adequate intake. The framework therefore separates the social value of an interaction from the nutritional appropriateness of its message, allowing both to be evaluated within a single episode.

### 3.1 Social connection and digital commensality

Physical eating arrangements provide a contextual foundation for the social-connection pathway. Among older Japanese adults living alone, infrequent shared meals were associated with lower food diversity or frequency and poorer self-rated health (Ishikawa et al., 2017). Eating alone was also associated with depression, lower quality of life, and lower food diversity and body mass index (Kimura et al., 2012). These associations connect the social organization of meals with affect and dietary variety.

Digital commensality encompasses distinct social experiences (Pereira-Castro et al., 2022; Spence et al., 2019). Synchronous, two-way shared meals offer real-time interaction; asynchronous exchanges of photographs, recipes, or messages maintain relationships through delayed feedback. Passive or parasocial viewing can provide perceived company with limited interpersonal reciprocity. Conversational agents supply a further, technologically mediated form of social presence. These differences specify which component of a digital encounter is expected to matter for eating.

Adjacent interventions help identify those components. FridgeNet combined nutrition monitoring, diet sharing, and social interaction in a small field evaluation (Lee et al., 2015). A randomized trial of embodied conversational agents found no significant improvement in fruit, vegetable, or fluid intake or loneliness; interviews described increased awareness and attempts to change eating (Kramer et al., 2022). A Facebook-supported, multicomponent lifestyle intervention reduced psychological distress among adults aged 60 and older, alongside substantial dropout (Mastrogiovanni et al., 2025). The latter finding concerns the intervention package and a psychological outcome; together, these studies motivate joint assessment of social experience and eating.

Socioemotional selectivity theory links shorter perceived future time horizons to greater priority for emotionally meaningful goals and relationships (Carstensen, 2021), directing attention to the continuity and personal meaning of contact. A familiar meal companion and an unfamiliar livestream audience can offer different forms of social presence despite similar contact duration. Proposition 1: relationship continuity and reciprocity predict meal regularity and dietary variety beyond contact frequency, particularly among people who otherwise eat alone.

Relationship meaning also helps explain engagement burden. Reciprocal exchanges require time, attention, and a willingness to disclose everyday eating; these demands can vary with fatigue, privacy preferences, and the availability of assistance. Assessing social presence, perceived obligation, and enjoyment alongside meals can distinguish the experience of relational support from simple contact frequency.

### 3.2 Food information and nutrition literacy

The direct user evidence centers on dietary self-management. In a cross-sectional study of 257 short-video users aged 60 and older with type 2 diabetes, content classified as highly beneficial for diabetes was associated with higher self-reported general-diet and overall self-management scores; reported viewing duration was nonlinearly associated with overall self-management (Ye et al., 2025). A broader Thai analysis linked a regional internet-use proxy to food variety across ages (Teerakapibal and Melanthiou, 2019). This is broader-age, proxy-exposure evidence. This contrast directs future tests toward content and individual engagement, measured separately from area-level digital access.

Content audits examine the information available for appraisal. YouTube nutrition videos aimed at older adults showed limitations in reliability, comprehensiveness, and accuracy, with higher quality among professional sources (Sözlü and Aslan, 2026). Diet and sarcopenia videos also varied in quality and practical usefulness (Trost et al., 2026). These studies concern published content, whereas the behavioral question is which messages users encounter, trust, understand, and apply. Source credibility and usable guidance are therefore distinct targets for audience research.

Heuristic–systematic processing offers a psychological account of credibility appraisal (Chaiken, 1980). Credentials, familiarity, and social endorsement can serve as quick judgment cues; assessing arguments and comparing sources involves more effortful processing. Applied to later-life nutrition, the model directs attention to health relevance and prior experience as reasons for scrutiny, and to presentation demands and interface accessibility as conditions affecting that scrutiny. Reviews associate older-adult digital health literacy with socioeconomic resources, devices, and social support, and identify gaps in how competencies are measured (Oh et al., 2021; Shi et al., 2024). Chronological age alone does not specify a user’s processing strategy.

Qualitative evidence also positions users as active interpreters. Interviews with 25 UK adults aged 18–25 described checking expertise, comparing information, and selectively engaging with or disengaging from food and nutrition content (Tang et al., 2026). These accounts provide a broader-age mechanism bridge for studying how older adults search, save, share, verify, or reject advice. Such actions can alter both the information encountered and its meaning, making content curation part of the pathway itself.

Proposition 2: source cues and presentation demands jointly shape credibility appraisal, comprehension, and food choice, with digital literacy and health complexity conditioning these relationships. Experiments can vary source disclosure and presentation load, then measure understanding and food decisions separately. Combining these tests with qualitative accounts would reveal when familiar sources facilitate sound judgment and when trust substitutes for checking a claim.

Applied literacy also includes translating an understood message into a feasible meal. Researchers can ask participants to explain how a recommendation fits their food budget, cooking arrangements, cultural preferences, and existing dietary advice. A person may correctly identify a credible source yet decide that its recommendation is unsuitable. Capturing that decision preserves informed refusal as an expression of agency rather than treating all non-adherence as failed communication.

### 3.3 Social comparison, affect, and dietary self-regulation

The comparison pathway connects food- and body-related content with appraisal, affect, and dietary regulation. Broader-age reviews associate online comparison with body-image concerns and eating-disorder symptoms and identify different relationships across content categories (Bonfanti et al., 2025; Wu et al., 2024). In adults older than 60, body dissatisfaction was associated with affective symptoms, coping, emotion regulation, and problematic social-media use, with gender-related differences (López-Montón et al., 2025). This older-adult psychological evidence identifies processes to connect with eating outcomes.

Later-life comparison can concern appearance, disease control, strength, or independence. These goals give similar messages different nutritional meanings. Restrictive diets or celebrated weight loss may reinforce inadequate intake in people with poor appetite, unintentional weight loss, or malnutrition risk. Geriatric nutrition guidance emphasizes adequate intake and individualized care (Volkert et al., 2022). The relevant psychological question is how users interpret food rules against their own goals and health circumstances. Maintaining energy and protein intake can be central for people at risk of sarcopenia or frailty, while clinically indicated weight management calls for a different evaluation of the same content.

This distinction changes what counts as a beneficial response to content. Increased willingness to eat a varied meal can be valuable when appetite is low, whereas avoiding an unnecessary food restriction can support adequacy without producing weight loss. Studies should record users’ intended goals and baseline nutritional risk, then assess goal attainment and nutritional adequacy separately. Muscle function and nutritional status are downstream outcomes; proximal tests should establish how the message changes appraisal, appetite, food choice, and intake.

Jiao (2024) examined mixed food-media exposure, negative emotions, and unhealthy eating in a Shanghai lockdown sample aged 50 and older; 94% were aged 50–60. The cross-sectional path analysis reported statistical indirect associations involving negative emotions, food literacy, health consciousness, and eating self-efficacy. In another sample aged 50–90, recorded social-media use over at least 2 weeks showed no significant association of social-media duration with nutritional status, loneliness, depression, or anxiety (Polatoz et al., 2026). This null duration finding concerns different endpoints from eating behavior and comparison appraisal.

Proposition 3: function-, appearance-, and disease-control comparisons precede within-person changes in affect and self-efficacy that relate to subsequent eating. Outcomes should include appetite, meal regularity, adequate intake, restrictive or emotional eating, and disease-specific adherence. For a person at nutritional risk, a comparison that encourages skipping meals differs from one that supports achievable, protein-containing meals. Repeated assessments can test both directions: distress or appetite loss may prompt content seeking, as well as follow particular digital encounters.

### 3.4 Commercial cues and algorithmic curation

The commercial pathway extends digital food-environment research into a later-life hypothesis about exposure and persuasion. Granheim et al. (2022) mapped digitalization across food marketing, information, availability, and price. A youth-focused review documented social-media marketing of ultra-processed products (Lafontaine et al., 2025). These sources establish the broader commercial context; the later-life proposition concerns how repeated messages, source cues, and purchasing opportunities relate to credibility and food decisions.

Platform environments differ in the balance of followed accounts, recommended content, paid advertisements, influencer sponsorship, livestreaming, and integrated shopping. These features should be coded separately because they combine different opportunities for attention, social endorsement, and immediate purchase. Later-life research can examine supplements, protein products, meal replacements, and disease-management claims alongside ordinary foods. Product categories should be identified from observed feeds, with attention to users’ health goals, financial resources, and capacity to evaluate provenance.

For example, a sponsored testimonial embedded in an otherwise supportive community combines interpersonal endorsement with a commercial source. A product link attached to an instructional video combines advice with an immediate purchasing opportunity. Comparing these formats would show whether persuasion depends on the source, repetition, perceived similarity to the presenter, or the ease of acting on the message.

The proposed mechanism is that repeated, personally relevant commercial messages increase familiarity and perceived credibility, which then inform food-related decisions. This connects commercial persuasion with the information-appraisal pathway. A health-related search followed by repeated product recommendations offers a test scenario for examining how curation changes exposure. Participant-authorized feed records and controlled profile audits can measure the sequence; interviews can show how users interpret, resist, or act on it. Credibility, purchase intention, actual purchasing, and eating represent separate stages requiring separate measures.

Proposition 4: repeated commercial exposure predicts claim credibility and food-related decisions, with source disclosure, digital health literacy, and health-goal relevance conditioning the relationship. Platform comparisons and experiments can distinguish repetition from message content and pre-existing user interests. The critical test is whether changes in audited exposure precede changes in appraisal and decisions among older users.

## 4 Discussion

### 4.1 Later-life moderators and digital inequalities

Chronological age indexes different life circumstances without specifying a single psychological profile. Studies beginning at 50 include transitions around employment and retirement; samples beginning at 60 or 65 capture other mixtures of health, relationships, and technology experience. Findings from predominantly younger, digitally confident participants have their clearest application to comparable users. Sampling adults aged 75 and older, people who need assistance, and those with multimorbidity would extend tests of the framework across later-life circumstances. Digital literacy and resources shape both participation and the feasibility of acting on nutrition information (Shi et al., 2024).

Age distributions should accompany eligibility thresholds because a lower age limit does not describe the composition of a sample. Jiao’s predominantly 50–60-years-old sample and the older diabetes users studied by Ye et al. (2025) represent different health and life-course settings. Reporting age, functional status, assistance needs, and technology experience together supports judgments about transferability and helps separate chronological-age differences from differences in resources or exposure.

Cognitive, sensory, and motor conditions affect navigation and comprehension in interaction with interface design. Frailty and chronic disease also shape the nutritional meaning of online advice. Social-media use was associated with frailty and sociality indicators in a Japanese cross-sectional survey (Nakada and Seo, 2025). This association identifies frailty as a candidate characteristic for prospective interaction tests, alongside oral health, medication burden, sensory function, and food assistance. Assessing these characteristics at baseline would help distinguish pre-existing nutritional vulnerability from subsequent dietary change.

Living arrangements and relationship quality shape whether online contact supplements offline support. Access to a valued meal companion differs from participation in a large, unfamiliar group, even when both involve regular messaging. Socioeconomic position affects devices, data plans, food affordability, and opportunities to follow advice. Culture shapes familiar foods, family roles, body norms, and trusted sources. Their intersections offer specific explanations for variation in pathway strength: an accessible message can still be impractical when recommended foods are unaffordable or preparation requires unavailable assistance.

### 4.2 Limitations and future directions

The focused narrative approach prioritizes integration across heterogeneous sources, leaving the completeness and methodological quality of direct older-adult evidence to be assessed. A full systematic review with prespecified eligibility criteria, comprehensive searches, documented record-level screening, and risk-of-bias appraisal, reported in accordance with PRISMA 2020, could assess the extent and quality of evidence for each pathway.

Exposure and agency. Studies should combine participant-authorized platform records with interviews, think-aloud tasks, or content-use diaries to examine how users find, evaluate, save, share, and avoid food information. Records should distinguish platform, source, commercial status, interaction mode, and timing relative to meals. Pairing these observations with meal regularity, dietary variety, appetite, intake, or validated dietary self-management measures connects psychological processes to eating. Nutritional status requires a longer observation window. Privacy safeguards should accommodate health information and third-party communications within digital traces.

Interview prompts can use participants’ voluntarily selected examples to examine why a post was saved, shared with family, checked elsewhere, or ignored. Linking those explanations to food diaries would connect interpretation with subsequent meals. This approach also captures changes in platform use after a diagnosis, a change in living arrangement, or difficulty maintaining an adequate diet.

Temporal and mechanism tests. Intensive longitudinal studies can examine whether exposure precedes changes in appraisal, affect, or meals within individuals, and whether eating difficulties prompt subsequent content seeking. Experiments can vary a specific relational or information feature while measuring its proposed mediator and eating response. Multicomponent interventions require component-level comparisons to identify what produces change. Longer follow-up can assess persistence, unintended restriction, and substitution for offline support. The pathway-specific comparisons in Table 1 provide testable starting points.

Inclusive interventions and platform research. Recruitment should extend beyond confident smartphone users through supported access, flexible participation, and clear reporting of technology-related nonparticipation (Goodwin et al., 2023). Co-design can identify meaningful social contact, understandable information, and feasible dietary actions. Feed audits should compare commercial formats and recommendation patterns, followed by tests of accessible controls and source disclosure. Combining these approaches would locate effective changes at the level of the user, the interaction, or the platform, while preserving autonomy and access to valued relationships.

## 5 Conclusion

Social-media research on later-life eating benefits from specifying content, engagement, and the circumstances in which meals occur. The four-pathway framework connects relationship continuity, credibility appraisal, comparison-related affect, and commercial persuasion with eating outcomes. Its distinctive contribution is to place emotionally meaningful relationships, active information use, and adequate nutrition within a common psychological account of later life. Source-level evidence mapping identifies where direct findings inform that account and where theory guides new tests. Research that measures these processes over time and includes diverse older users can determine which digital experiences support appropriate, sufficient, and enjoyable eating.
