# Supplementary material for: Social media and eating behavior in later life: psychological pathways, evidence gaps, and an age-inclusive research agenda
Source: Front Psychol. 2026 Oct 12;17:1966068. doi: 10.3389/fpsyg.2026.1966068 (PMC13696252; doi:10.3389/fpsyg.2026.1966068)
Supplement: Supplementary file 1 [file Supplementary_file_1.docx]

Supplementary Material S1

**Search record and source-level evidence map for Social Media and Eating Behavior in Later Life**

# Search scope and source selection

The focused narrative review integrates social-media and eating research with later-life eating contexts. Its main interpretive population is adults aged 60 years and older; studies beginning at age 50 are retained as mid-to-later-adulthood evidence. Source selection prioritizes relevance to a specified psychological pathway or a cross-cutting later-life condition. General internet measures, mixed-media exposure, conversational agents, and multicomponent digital-health interventions are identified by their measured exposure rather than treated as interchangeable with social-media use. Social media used only for recruitment or data collection does not constitute exposure evidence.

Database discovery took place on 12 August 2026 in Web of Science Core Collection and Scopus. Retained records were restricted to English-language articles and reviews published in 2004–2026; some restrictions were applied after export. The primary concept groups were older age, social media or named platforms, and eating, food, or nutrition. Broader life-course searches informed mechanism-bridging source identification. The retained expressions are reproduced below.

## Retained database expressions

## Web of Science initial older-age intersection

("older adult*" OR "older people" OR "older person*" OR "older population*" OR elderl* OR geriatric* OR "senior citizen*" OR "later life" OR "late life" OR "old age")
AND
("social media" OR "social networking site*" OR "online social network*" OR "social network platform*" OR Facebook OR Instagram OR YouTube OR TikTok OR Twitter OR WeChat OR WhatsApp OR "online communit*" OR "virtual communit*")
AND
("eating behavio?r*" OR "dietary behavio?r*" OR eat* OR diet* OR food* OR nutrition* OR meal* OR appetite OR cook* OR "food choice*" OR "food consumption")

Field: Topic. The initial query returned 152 records. The project log subsequently records an expansion using digital-health terms near social-support or interaction concepts (NEAR/5), with 175 records exported after article/review and English restrictions. The exact expanded Boolean expression was not retained; the expression above is the initial query. The 175-record export is preserved in the research archive.

## Scopus older-age intersection

TITLE-ABS-KEY(
 ("older adult*" OR "older people" OR "older person*" OR "older population*" OR elderl* OR geriatric* OR "senior citizen*" OR "later life" OR "late life" OR "old age")
 AND
 ("social media" OR "social networking site*" OR "online social network*" OR "social network platform*" OR Facebook OR Instagram OR YouTube OR TikTok OR Twitter OR WeChat OR WhatsApp OR "online communit*" OR "virtual communit*")
 AND
 ("eating behavio?r*" OR "dietary behavio?r*" OR eat* OR diet* OR food* OR nutrition* OR meal* OR appetite OR cook* OR "food choice*" OR "food consumption")
)

Field: TITLE-ABS-KEY. Recorded restrictions: 2004–2026, Article/Review, journal sources. The export contained 272 records; English eligibility was also checked during subsequent processing.

## Scopus broader life-course discovery

TITLE-ABS-KEY(
 ("social media" OR facebook OR instagram OR tiktok OR reddit OR wechat)
 AND
 ("eating behavior*" OR "eating behaviour*" OR "dietary behavior*" OR "dietary behaviour*"
 OR "food choice*" OR "food consumption" OR "dietary intake" OR "diet quality"
 OR "healthy eating" OR "food marketing" OR mukbang)
)
AND PUBYEAR > 2003 AND PUBYEAR < 2027
AND (DOCTYPE(ar) OR DOCTYPE(re)) AND SRCTYPE(j)

The complete export contained 1,952 records, of which 1,898 satisfied the recorded language, year, and document-type restrictions.

## Web of Science broader life-course coverage subset

TS=(("social media" OR Facebook OR Instagram OR TikTok OR Reddit OR WeChat)
AND ("eating behavio?r*" OR "dietary behavio?r*" OR "food choice*" OR "food consumption"
OR "dietary intake" OR "diet quality" OR "healthy eating" OR "food marketing" OR mukbang))

The query returned 1,269 records. The first 500 records ordered by relevance were exported as a coverage and metadata subset; 474 met subsequent restrictions.

## Discovery records and targeted supplementation

The older-age exports contained 447 records; 438 met the recorded metadata restrictions and 332 remained after within-pool deduplication. The broader life-course exports contained 2,452 records; 2,372 met the recorded metadata restrictions and 1,959 remained after within-pool deduplication. Supplementary Figure S1 presents these discovery stages alongside the composition of the 26 synthesis sources and five background references.

On 5 September 2026, targeted title and DOI retrieval added Tang et al. (2026), identified through the reviewer’s recommendation, and sources on socioemotional selectivity (Carstensen, 2021), heuristic–systematic processing (Chaiken, 1980), and geriatric nutritional priorities (Volkert et al., 2022). Foundational theory was selected independently of the database date window. Tang et al. was published on 7 April 2026. This supplementary retrieval addressed the specified conceptual and qualitative questions rather than repeating the original database searches.

**Supplementary Figure S1.** Database discovery and cited-source composition for the focused narrative review. The two database pools are displayed separately because they can overlap. Filtered denotes the recorded metadata restrictions. The 175 Web of Science records are the archived expanded export; the retained initial query returned 152. The broader Web of Science search returned 1,269 records, of which the first 500 ranked by relevance were exported. The cited-source set also draws on targeted supplementary identification and is displayed separately from the discovery-stage counts.

## Evidence classes and counting convention

D denotes studies evaluating a defined social-media exposure in relation to an eating behavior among older users, including dietary choice, intake, variety, regularity, or dietary self-management. C denotes later-life eating-context evidence without social-media exposure. T denotes older-targeted or later-life-relevant content audits. A denotes adjacent mid-to-later-adulthood evidence involving mixed media, digital-health technologies, or psychological and nutritional-status endpoints. Multicomponent digital-health evaluations are A when their social-media contribution to eating outcomes is not separately evaluated. M denotes broader-age mechanism or contextual evidence; G denotes youth-focused evidence used primarily to identify an age gap. The primary role is assigned according to the source’s use in this synthesis: a younger-population study can supply a mechanism bridge (M) or serve as an age-gap comparator (G).

There are 31 reference sources, of which 26 enter the six-class synthesis: D=1, C=5, T=2, A=10, M=7, and G=1. These comprise 15 primary investigations (nine cross-sectional studies, two randomized trials, one uncontrolled field evaluation, two content analyses, and one qualitative interview study), ten systematic or scoping reviews, and one conceptual review. The remaining five references supply demographic background, theory, guidance, or research-design recommendations.

Table 1 in the main manuscript counts sources by their primary section: social connection, 7 (C=2, A=3, M=2); food information, 7 (D=1, T=2, A=2, M=2); comparison, 5 (A=3, M=2); commercial cues, 2 (M=1, G=1). Five further sources support the introduction or cross-cutting conditions (C=3, A=2). Thus 7+7+5+2+5=26 unique sources. Secondary relevance to another pathway does not add to these counts. Participant numbers and studies contained within reviews are not pooled. Evidence classes describe roles; study design and measurement determine inferential strength.

# Supplementary Table S1 Source-level evidence map

Sources are grouped by primary manuscript section. Sample sizes refer to the units reported in each source. “Not reported” or “not extracted” identifies a source-level reporting or retrieval limit, not a zero value. The primary class appears with each citation; the five sources outside the synthesis denominator follow the 26 synthesis sources.

| **Source and primary role** | **Population and sample** | **Study design** | **Exposure or topic** | **Measured outcome and contribution** |
| --- | --- | --- | --- | --- |
| Ishikawa et al., 2017 C 3.1[· DOI](https://doi.org/10.1007/s12603-016-0805-z) | 65–90 2196 participants Seven Japanese towns/cities; living alone | Cross-sectional | Frequency of eating together | Food diversity/frequency; self-rated health; meal satisfaction Among older Japanese adults living alone, infrequent shared meals were associated with poorer self-rated health and lower food diversity/frequency. |
| Kimura et al., 2012 C 3.1[· DOI](https://doi.org/10.1007/s12603-012-0067-3) | >=65; mean 76.6 (SD 7.8) 856 participants Tosa, Japan; community dwelling | Cross-sectional | Eating alone and living arrangements | Food diversity; depression; quality of life; BMI Eating alone was associated with depression, lower quality of life, and lower food diversity and body mass index in older Japanese adults. |
| Kramer et al., 2022 A 3.1[· DOI](https://doi.org/10.2196/33974) | >=65; mean 73.0 (SD 5.33), range 65–85 in 32 users 51 met eligibility criteria; 32 completed the intervention Netherlands; Dutch-speaking, retired, independently living alone | Randomized trial | PACO web intervention with embodied conversational agents and self-monitoring | Fruit/vegetable/fluid intake; loneliness; use; perceived usefulness and experiences The trial reported no significant improvement in measured intake or loneliness; interviews described increased dietary awareness and differing engagement preferences. |
| Lee et al., 2015 A 3.1[· DOI](https://doi.org/10.1109/mis.2015.55) | 55–76; mean 66.27 (SD 6.13) 15 living-alone participants; 12-week evaluation Field location not explicitly stated in the article | Uncontrolled field evaluation | FridgeNet bundle: sensor-linked dietary tracking, nutrition feedback, peer recommendations/comments and shopping invitations | Logged nutrient-insufficiency counts; viewing/likes/comments; shopping invitations; user feedback A 12-week FridgeNet field evaluation recorded dietary monitoring and peer interaction in a small older-user sample. |
| Mastrogiovanni et al., 2025 A 3.1[· DOI](https://doi.org/10.1093/ageing/afaf190) | >=60; mean age not available in the retained extraction 80 randomized participants Australia; independently living, English-speaking, technology-enabled participants | Randomized trial | MovingTogether: private Facebook group plus exercise, StandingTall and lifestyle education | Psychological distress and other non-dietary secondary outcomes; interviews A Facebook-supported lifestyle intervention reduced psychological distress but not measured secondary outcomes and did not establish dietary improvement. |
| Pereira-Castro et al., 2022 M 3.1[· DOI](https://doi.org/10.3390/ijerph192416734) | No later-life restriction; study-specific ages 104 studies Multiple countries; no single national sample | Scoping review | Digital forms of commensality | Six forms of digitally mediated eating/social interaction Digital commensality includes remote shared meals, solo technology use, virtual food communities, and commercial forms. |
| Spence et al., 2019 M 3.1[· DOI](https://doi.org/10.3389/fpsyg.2019.02252) | Life-course conceptual coverage; no single age criterion Not a fixed empirical sample Not applicable; conceptual review | Conceptual review | Technology-mediated eating/drinking companionship | Conceptual forms and possible mechanisms of digital commensality Digital commensality can be conceptualized as technology-mediated social eating rather than mere simultaneous screen use. |
| Oh et al., 2021 A 3.2[· DOI](https://doi.org/10.2196/26145) | Study-defined older adults; common thresholds >=50 or >=60 27 studies Multiple countries, predominantly USA | Systematic review | Digital literacy measurement among older adults | Coverage of digital competence dimensions and instrument properties Measures used in older-adult digital-literacy research often cover only a limited subset of competence dimensions. |
| Shi et al., 2024 A 3.2[· DOI](https://doi.org/10.3389/fpubh.2024.1447747) | Study-defined older adults; heterogeneous thresholds 28 articles Multiple countries, principally China and USA | Scoping review | Factors associated with digital health literacy | Digital health literacy, demographic and social factors Older-adult digital health literacy varies with sociodemographic, device-related, and social-support factors. |
| Sözlü & Aslan, 2026 T 3.2[· DOI](https://doi.org/10.1080/03601277.2025.2573393) | Videos targeted to older audiences; no participant age 49 eligible videos from 1001 initially identified YouTube content; audience country unknown | Content analysis | Older-adult nutrition videos; source type | GQS; modified DISCERN; geriatric nutrition scoring YouTube nutrition videos aimed at older adults frequently lacked reliability, comprehensiveness, or accuracy despite better quality from professional sources. |
| Tang et al., 2026 M 3.2[· DOI](https://doi.org/10.2196/89344) | 18–25; mean 22.2 (SD 1.9) 25 analyzed participants United Kingdom; English-speaking regular social-media users | Qualitative interviews | Experiences with food and nutrition content on social media | Credibility appraisal; active engagement; content curation and protective responses Young adults described evaluating credibility and actively managing their food-content exposure, providing mechanism-bridging accounts of user agency. |
| Teerakapibal & Melanthiou, 2019 M 3.2[· DOI](https://doi.org/10.1108/bfj-08-2018-0559) | 6–98; mean 40.4 (SD 20.2) 66556 complete records Thailand; merged 2013 national Health and Wellness and Internet Usage surveys | Cross-sectional | Region-by-municipality proportion using internet, an ecological proxy, not individual social-media exposure | Count of food types consumed at least weekly; chronic disease status A Thai population analysis associated an area-level internet-use proxy with food variety, illustrating an adjacent digital-access question. |
| Trost et al., 2026 T 3.2[· DOI](https://doi.org/10.3390/nu18040619) | Sarcopenia/nutrition relevance; no observed audience ages 41 eligible videos from 60 screened English-language YouTube search, April 2024; audience geography unknown | Content analysis | YouTube sarcopenia diet content; default relevance ranking | Quality, reliability, clinical content, advertising and potential hazards YouTube videos about diet for sarcopenia showed variable quality and often vague or impractical guidance for older adults. |
| Ye et al., 2025 D 3.2[· DOI](https://doi.org/10.2196/67312) | >=60; median 69 (IQR 65–72) 257 valid questionnaires from 268 recruited Jinan, China; five community health centers; type 2 diabetes | Cross-sectional | Short-video app time and content; reports supplemented with use/history records | Diabetes self-management including general diet and specific diet domains Highly beneficial diabetes content was cross-sectionally associated with better self-reported general-diet and overall self-management scores; reported viewing time showed a nonlinear association with overall self-management. |
| Bonfanti et al., 2025 M 3.3[· DOI](https://doi.org/10.1016/j.bodyim.2024.101841) | Broad-age correlational literature; older-specific subset not established 83 studies; 55440 participants reported in abstract Multiple countries | Systematic review and meta-analysis | Social comparison on social media | Body-image concerns; positive body image; eating-disorder symptoms Online social comparison is associated with body-image concerns and eating-disorder symptoms in the broader literature. |
| Jiao, 2024 A 3.3[· DOI](https://doi.org/10.3390/foods13121797) | >=50; range 50–81; mean 53.66 (SD 4.08); 94% age 50–60 400 valid respondents Shanghai, China; COVID-19 Omicron lockdown context | Cross-sectional | 12-source composite food-media exposure including cookbooks, print, television and social media | Unhealthy eating; negative emotions; food literacy and eating self-efficacy Mixed food-media exposure showed statistical indirect associations with unhealthy eating through negative emotions in a predominantly 50–60-year-old sample. |
| López-Montón et al., 2025 A 3.3[· DOI](https://doi.org/10.1016/j.paid.2025.113282) | >60; mean age not available in the accessible report preview 736 participants Spain | Cross-sectional | Problematic social-media use; emotion regulation, distress, support, coping | Body dissatisfaction Body dissatisfaction in adults older than 60 showed gender-differentiated associations with affective, coping, emotion-regulation, and problematic-social-media factors. |
| Polatoz et al., 2026 A 3.3[· DOI](https://doi.org/10.1002/hsr2.72352) | >=50; range 50–90; mean 64.2 (SD 9.7) 153 participants Turkish-language clinical/community survey; specific field site not available in the retained extraction | Cross-sectional | Objective smartphone screen-time records (>=2 weeks) and reported social-media duration | Mini Nutritional Assessment; loneliness; depression; anxiety Objectively recorded and self-reported social-media duration were not significantly associated with nutritional status, loneliness, depression, or anxiety among adults aged 50 years or older. |
| Wu et al., 2024 M 3.3[· DOI](https://doi.org/10.1016/j.bodyim.2023.101650) | Broad-age samples, mainly younger; no older-specific synthesis 38 studies Multiple countries | Systematic review | Eating-related social-media content including food promotion and eating videos | Body image; eating behavior; viewer perceptions Different categories of social-media food content show different relationships with body image, disordered eating, and consumption-related outcomes. |
| Granheim et al., 2022 M 3.4[· DOI](https://doi.org/10.1111/obr.13356) | No later-life restriction 357 studies Multiple countries; digital food environment | Scoping review | Digital food environment across marketing, access and other dimensions | Food environment features and reported dietary/health evidence Digitalization can affect multiple dimensions of food environments, including marketing, availability, prices, and information. |
| Lafontaine et al., 2025 G 3.4[· DOI](https://doi.org/10.1136/bmjgh-2023-014667) | Children/adolescents 0–18 36 articles Multiple, mostly high-income countries | Scoping review | Commercial social-media industry/digital food environment | Food advertising exposure; awareness; consumption/behavior; regulation Commercial social-media food-environment research has concentrated on children and adolescents and commonly documents exposure to ultra-processed-food advertising. |
| Kim, 2016 C Introduction / shared context[· DOI](https://doi.org/10.1016/j.appet.2015.09.015) | Mean 78.6 (SD 5.7); lower inclusion cutoff not available in the accessible report preview 87 participants Seoul, South Korea; low-income frail home-healthcare recipients | Cross-sectional | Offline social network type and food choice values | Mean adequacy ratio/diet quality Social networks are relevant to food choice and diet quality among frail older adults. |
| Montez De Sousa et al., 2022 C Introduction / shared context[· DOI](https://doi.org/10.3389/phrs.2022.1604967) | Study-defined older adults; youngest included threshold 50 77 publications High-income countries; community-dwelling older adults | Scoping review | Ecological determinants of eating | Eating behavior and food choice Older-adult eating behavior is influenced across individual, interpersonal, community, and societal levels. |
| Nakada & Seo, 2025 A Introduction / shared context[· DOI](https://doi.org/10.3390/ijerph22020142) | >=65 103 main-survey respondents Sumida Ward, Tokyo and Matsudo City, Japan | Cross-sectional | Reported social-media engagement | Kihon Checklist frailty and associated psychological/social variables Social-media engagement was associated with frailty and sociality indicators in older Japanese adults. |
| Newman et al., 2021 A Introduction / shared context[· DOI](https://doi.org/10.1017/s0144686x19001144) | Mean sample age >=65 in each paper; participant range 50–98 21 papers Multiple countries; country distribution not available in the retained extraction | Systematic review | Social-networking-site use and user experience | Motivations; barriers; wellbeing; cognition Older adults often use social media to maintain relationships, while privacy, relevance, and usability can impede participation. |
| Walker-Clarke et al., 2022 C Introduction / shared context[· DOI](https://doi.org/10.1016/j.arr.2022.101597) | Eligibility >60 years 53 articles Multiple countries; predominantly Western/community settings | Systematic review | Psychological and social influences on eating | Eating attitudes; intake; dietary quality; nutritional outcomes Later-life eating is shaped by health beliefs, control, mood, eating arrangements, social facilitation, and support. |
| Carstensen, 2021 Outside synthesis count Background / theory / guidance[· DOI](https://doi.org/10.1093/geront/gnab116) | Life-span theory; not a single late-life sample Not a primary sample Not applicable | Theoretical review | Perceived time horizons and meaningful goals | Motivational priorities and socioemotional selectivity Perceived endings can prioritize emotionally meaningful goals, motivating attention to relationship quality and continuity. |
| Chaiken, 1980 Outside synthesis count Background / theory / guidance[· DOI](https://doi.org/10.1037/0022-3514.39.5.752) | Undergraduate experiments; exact age range not reported in participant descriptions Experiment 1: 207 initially, before exclusions; Experiment 2: 80; a separate 125-person group supplied control opinions United States and Canada; University of Massachusetts and University of Toronto | Theory foundation experiments | Source likability and number of arguments under differing involvement | Persuasion/opinion change Persuasion can reflect source cues or more effortful consideration of message arguments, depending on involvement. |
| Goodwin et al., 2023 Outside synthesis count Background / theory / guidance[· DOI](https://doi.org/10.1093/ageing/afad082) | Older people; no single cutoff Not a dietary-effect sample UK-led recommendations; general research design | Framework based recommendations | INCLUDE-based participation and research access | Inclusive research design recommendations Inclusive ageing research should avoid upper age limits, accommodate multimorbidity and cognitive impairment, and offer supported or non-digital participation routes. |
| Volkert et al., 2022 Outside synthesis count Background / theory / guidance[· DOI](https://doi.org/10.1016/j.clnu.2022.01.024) | Older persons, generally >=65 82 recommendations, not a study sample International ESPEN clinical guideline | Evidence based consensus guideline | Clinical nutrition and hydration priorities | Malnutrition prevention; intake adequacy; individualized weight management Age-related appetite decline and inadequate intake make nutritional sufficiency and preservation of function important priorities; weight-reducing approaches require individualized indications. |
| World Health Organization, 2025 Outside synthesis count Background / theory / guidance | Population projections for >=60 years Not an included empirical study Global | Official demographic background | Population ageing | Demographic projections and ageing context By 2030, one in six people worldwide will be aged 60 years or older; this population is projected to reach 2.1 billion by 2050. |

Source-specific interpretation: Jiao (2024) combines several food-media sources, including non-social media; Teerakapibal and Melanthiou (2019) use regional internet prevalence as a proxy in a cross-age sample. Lee et al. (2015) evaluate a multicomponent monitoring and social-support system. Ye et al. (2025) report a self-reported general-diet domain within diabetes self-management. Content audits characterize available material, not observed audience response.
